# Supplementary material for: Abrupt light transitions in illuminance and correlated colour temperature result in different temporal dynamics and interindividual variability for sensation, comfort and alertness
Source: PLoS One. 2021 Mar 22;16(3):e0243259. doi: 10.1371/journal.pone.0243259 (PMC7984641; doi:10.1371/journal.pone.0243259)
Supplement: S1 Table — Variances of the random intercept models. (PDF) [file pone.0243259.s001.pdf]

S2. Null model statistics

Table S2 Variances of random intercept models (not including random slopes)

Dependent variable	Residual variance [%]	ICC Session [%]	ICC Participant [%]
$Sensation_{VI}$	15.29	84.71	0.00
$Sensation_{VC}$	20.36	79.64	0.00
$Comfort_{V}$	22.12	51.01	26.87
Vitality	38.12	36.15	25.73
Sleepiness (KSS)	32.29	45.73	21.98
Mean RT (PVT)	10.98	10.33	78.69
Effort PVT	23.51	31.91	55.48
Correct (BDST)	22.41	11.33	66.26
Effort BDST	22.95	39.91	37.14
Mean SCL	4.16	30.98	64.86
Mean HR	13.01	23.80	63.20
Mean HRV	10.91	41.32	47.78
Calm	20.33	22.63	57.04
Happy	21.85	45.78	32.36
$Sensation_{T}$	47.58	38.33	14.09
Self-assessed shivering	29.89	33.00	37.11
$Comfort_{T}$	39.48	25.55	34.97
T_{skin}	12.02	50.57	37.41
DPG	2.55	97.45	0.00
