# Supplementary material for: Abrupt light transitions in illuminance and correlated colour temperature result in different temporal dynamics and interindividual variability for sensation, comfort and alertness
Source: PLoS One. 2021 Mar 22;16(3):e0243259. doi: 10.1371/journal.pone.0243259 (PMC7984641; doi:10.1371/journal.pone.0243259)
Supplement: S2 Table — F-statistic and p-vales of the covariates, full model fit and R2 of the random intercept models. (PDF) [file pone.0243259.s002.pdf]

S3. Full model statistics

Table S3 Full model statistics of random intercept models (not including random slopes) with F-statistics (F) and p-values (p) for the covariates, full model fit, R^2 and marginal R^2 referring to the R^2 explained by the fixed part.

Dependent variable	Time of day		Reading effort		Baseline		Illuminance x CCT x Block		Full model fit	R^2	R^2 fixed effects
	F	p	F	p	F	p	F	P			
Sensation$_{VI}$	$F_{1,21}$ = 0.00	0.98	$F_{1,249}$ = 2.60	0.11	$F_{1,80}$ = 2.52	0.12	$F_{2,182}$ = 0.01	0.99	$F_{14,186}$ = 41.45	0.88	0.54
Sensation$_{VC}$	$F_{1,23}$ = 0.31	0.58	$F_{1,246}$ = 0.80	0.37	$F_{1,81}$ = 1.43	0.24	$F_{2,181}$ = 0.40	0.67	$F_{14,186}$ = 24.63	0.83	0.44
Comfort$_V$	$F_{1,22}$ = 7.83	0.01	**$F_{1,246}$ = 12.05**	**< 0.001**	**$F_{1,73}$ = 8.11**	**< 0.01**	$F_{2,183}$ = 0.37	0.69	$F_{14,186}$ = 12.41	0.80	0.27
Vitality	$F_{1,18}$ = 0.50	0.49	**$F_{1,256}$ = 44.50**	**< 0.001**	**$F_{1,90}$ = 30.16**	**< 0.001**	$F_{2,182}$ = 0.57	0.57	$F_{14,186}$ = 15.37	0.68	0.39
Sleepiness (KSS)	$F_{1,21}$ = 1.96	0.18	**$F_{1,250}$ = 32.06**	**< 0.001**	**$F_{1,97}$ = 15.66**	**< 0.001**	$F_{2,183}$ = 0.52	0.60	$F_{14,186}$ = 13.27	0.73	0.33
Mean RT (PVT)	$F_{1,15}$ = 0.07	0.80	$F_{1,225}$ = 1.37	0.24	**$F_{1,81}$ = 66.41**	**< 0.001**	$F_{2,152}$ = 0.77	0.46	$F_{14,167}$ = 21.09	0.85	0.39
Effort PVT	$F_{1,17}$ = 0.48	0.50	**$F_{1,299}$ = 37.06**	**< 0.001**	**$F_{1,88}$ = 24.33**	**< 0.001**	$F_{2,172}$ = 1.57	0.21	$F_{14,186}$ = 15.79	0.72	0.37
Correct (BDST)	$F_{1,19}$ = 0.30	0.59	$F_{1,259}$ = 1.01	0.32	**$F_{1,83}$ = 41.59**	**<0.001**	$F_{2,183}$ = 0.89	0.41	$F_{14,186}$ = 9.64	0.72	0.22
Effort BDST	$F_{1,21}$ = 0.09	0.76	$F_{1,240}$ = 5.50	0.02	**$F_{1,94}$ = 191.31**	**<0.001**	$F_{2,182}$ = 1.01	0.37	$F_{14,186}$ = 33.22	0.75	0.59
Mean SCL	$F_{1,24}$ = 1.91	0.18	$F_{1,233}$ = 4.34	0.04	**$F_{1,69}$ = 465.50**	**< 0.001**	$F_{2,164}$ = 0.17	0.84	$F_{14,177}$ = 213.77	0.97	0.87
Mean HR	$F_{1,23}$ = 0.84	0.37	$F_{1,107}$ = 4.90	0.03	**$F_{1,38}$ = 949.98**	**< 0.001**	$F_{2,167}$ = 1.29	0.28	$F_{14,177}$ = 104.03	0.87	0.85
Mean HRV	$F_{1,86}$ = 0.92	0.34	$F_{1,247}$ = 2.50	0.12	**$F_{1,87}$ = 251.30**	**< 0.001**	$F_{2,169}$ = 0.32	0.73	$F_{14,177}$ = 77.09	0.92	0.71
Calm	$F_{1,22}$ = 0.30	0.59	F1,272 = 0.11	0.74	**$F_{1,94}$ = 45.26**	**< 0.001**	$F_{2,183}$ = 4.94	0.01	$F_{14,186}$ = 14.60	0.78	0.30
Happy	$F_{1,20}$ = 0.00	0.95	$F_{1,245}$ = 1.30	0.26	**$F_{1,88}$ = 54.58**	**< 0.001**	$F_{2,180}$ = 2.43	0.09	$F_{14,186}$ = 18.70	0.79	0.39
Sensation$_T$	$F_{1,21}$ = 0.39	0.54	$F_{1,229}$ = 1.20	0.28	**$F_{1,87}$ = 16.60**	**< 0.001**	$F_{2,181}$ = 0.22	0.80	$F_{14,186}$ = 5.14	0.58	0.17
Self-assessed shivering	$F_{1,23}$ = 2.10	0.16	$F_{1,272}$ = 0.94	0.33	**$F_{1,75}$ = 13.47**	**< 0.001**	$F_{2,177}$ = 0.20	0.82	$F_{14,186}$ = 6.45	0.72	0.17
Comfort$_T$	$F_{1,22}$ = 1.20	0.28	**$F_{1,266}$ = 10.33**	**< 0.001**	$F_{1,84}$ = 6.61	0.01	$F_{2,179}$ = 0.71	0.49	$F_{14,186}$ = 4.51	0.61	0.14
T_{skin}	$F_{1,24}$ = 0.01	0.92	$F_{1,256}$ = 0.21	0.64	**$F_{1,93}$ = 805.83**	**< 0.001**	$F_{2,181}$ = 0.74	0.48	$F_{14,185}$ = 298.36	0.97	0.89
DPG	$F_{1,22}$ = 0.44	0.52	$F_{1,220}$ = 0.36	0.55	**$F_{1,88}$ = 1852.33**	**< 0.001**	$F_{2,183}$ = 2.76	0.07	$F_{14,186}$ = 490.70	0.98	0.94
