# Supplementary material for: Abrupt light transitions in illuminance and correlated colour temperature result in different temporal dynamics and interindividual variability for sensation, comfort and alertness
Source: PLoS One. 2021 Mar 22;16(3):e0243259. doi: 10.1371/journal.pone.0243259 (PMC7984641; doi:10.1371/journal.pone.0243259)
Supplement: S3 Table — Delta, t-ratio and p-value for the main effect of illuminance and CCT separately. (PDF) [file pone.0243259.s003.pdf]

S4. Contrast estimates for main effects

Table S4 Contrast estimates for main effects for Illuminance and CCT separately

Dependent variable	CCT: Cool vs. Warm			Illuminance: Bright vs. Dim		
	Δ	t ratio	p-value	Δ	t ratio	p-value
$Sensation_{VI}$	0.28	2.01	0.048	**1.63**	**11.76**	**<0.001**
$Sensation_{VC}$	**-1.53**	**-9.23**	**<0.001**	0.00	0.00	1.00
$Comfort_{V}$	**-0.36**	**-2.72**	**<0.01**	-0.07	-0.55	0.59
Vitality	0.21	0.67	0.51	**0.99**	**3.19**	**<0.01**
Sleepiness (KSS)	-0.10	-0.41	0.68	**-0.65**	**-2.79**	**<0.01**
Mean RT (PVT)	4.67	0.82	0.42	-3.36	-0.59	0.56
Effort PVT	-0.11	-0.20	0.84	-0.7	-1.26	0.21
Correct (BDST)	0.08	0.38	0.71	0.00	0.00	1.00
Effort BDST	-0.11	-0.24	0.81	-1.11	-2.57	0.01
Mean SCL	-0.15	-0.79	0.43	0.03	0.15	0.88
Mean HR	0.83	1.28	0.20	-0.81	-1.26	0.21
Mean HRV	-3.43	-1.33	0.19	2.40	0.92	0.36
Calm	-0.08	-1.11	0.27	0.03	0.39	0.70
Happy	-0.20	-1.74	0.09	0.06	0.55	0.59
$Sensation_{T}$	0.06	0.44	0.66	0.08	0.59	0.56
Self-assessed shivering	-0.29	-1.15	0.25	-0.03	-0.12	0.90
$Comfort_{T}$	0.22	1.79	0.08	0.17	1.35	0.18
T_{skin}	0.05	1.10	0.27	-0.03	-0.64	0.53
DPG	0.04	0.21	0.84	0.19	1.06	0.29
