# Supplementary material for: Abrupt light transitions in illuminance and correlated colour temperature result in different temporal dynamics and interindividual variability for sensation, comfort and alertness
Source: PLoS One. 2021 Mar 22;16(3):e0243259. doi: 10.1371/journal.pone.0243259 (PMC7984641; doi:10.1371/journal.pone.0243259)
Supplement: S4 Table — Delta, t-ratio and p-value per measurement block for the effect of illuminance and CCT separately. (PDF) [file pone.0243259.s004.pdf]

S5. Contrast estimates per measurement block

Table S5 Contrast estimates per measurement block for Illuminance and CCT separately

Dependent variable	Block	CCT: Warm vs Cool			Illuminance: Bright vs. Dim		
		Δ	t-ratio	p-value	Δ	t-ratio	p-value
$Sensation_{VI}$	1	0.30	1.93	0.06	**1.96**	**12.53**	**< 0.001**
	2	0.25	1.59	0.11	**1.53**	**9.73**	**< 0.001**
	3	0.29	1.82	0.07	**1.41**	**8.99**	**< 0.001**
$Sensation_{VC}$	1	**-2.03**	**-10.82**	**< 0.001**	-0.06	-0.32	0.75
	2	**-1.32**	**-7.03**	**< 0.001**	0.04	0.22	0.82
	3	**-1.24**	**-6.61**	**< 0.001**	0.02	0.11	0.91
$Comfort_{V}$	1	**-0.53**	**-3.59**	**< 0.001**	-0.15	-1.00	0.32
	2	-0.37	-2.49	0.014	-0.10	-0.67	0.50
	3	-0.18	-1.18	0.24	0.03	0.21	0.83
Vitality	1	-0.04	-0.09	0.93	**1.34**	**3.33**	**< 0.01**
	2	0.08	0.20	0.84	**1.11**	**2.76**	**< 0.01**
	3	0.58	1.44	0.15	0.52	1.29	0.20
Sleepiness (KSS)	1	0.08	0.30	0.76	**-0.82**	**-2.93**	**< 0.01**
	2	-0.03	-0.09	0.93	**-0.78**	**-2.77**	**< 0.01**
	3	-0.34	-1.22	0.22	-0.34	-1.21	0.23
Mean RT (PVT)	1	14.41	1.96	0.05	-3.02	-0.41	0.68
	2	5.77	0.79	0.43	-2.19	-0.30	0.77
	3	-6.18	-0.84	0.40	-4.86	-0.66	0.51
Effort PVT	1	0.16	0.22	0.83	-1.51	-2.08	0.04
	2	0.28	0.39	0.70	-0.32	-0.44	0.66
	3	-0.77	-1.07	0.29	-0.28	-0.39	0.70
Correct (BDST)	1	0.39	1.36	0.18	-0.17	-0.59	0.56
	2	-0.13	-0.46	0.65	0.16	0.57	0.57
	3	-0.02	-0.08	0.94	0.00	0.00	1.00
Effort BDST	1	-0.02	-0.03	0.98	-0.46	-0.74	0.46
	2	-0.16	-0.26	0.80	-1.07	-1.70	0.09
	3	-0.14	-0.23	0.82	**-1.81**	**-2.88**	**< 0.01**
Mean SCL	1	-0.03	-0.14	0.89	0.23	1.07	0.29
	2	-0.26	-1.22	0.23	-0.09	-0.42	0.68
	3	-0.16	-0.73	0.46	-0.05	-0.25	0.80
Mean HR	1	-0.11	-0.11	0.91	0.22	0.22	0.82
	2	0.69	0.69	0.49	-1.14	-1.15	0.25
	3	1.91	1.91	0.06	-1.51	-1.51	0.13
Mean HRV	1	-3.18	-1.12	0.27	2.91	1.01	0.31
	2	-5.13	-1.79	0.08	1.87	0.65	0.52
	3	-1.99	-0.70	0.49	2.44	0.84	0.40
Calm	1	-0.02	-0.23	0.82	0.06	0.65	0.52
	2	-0.09	-0.98	0.33	-0.05	-0.53	0.60
	3	-0.13	-1.41	0.16	0.08	0.81	0.42
Happy	1	**-0.36**	**-2.76**	**< 0.01**	-0.01	-0.11	0.92
	2	-0.15	-1.15	0.25	0.20	1.51	0.13
	3	-0.08	-0.58	0.56	0.00	0.01	0.99
$Sensation_{T}$	1	0.07	0.42	0.68	0.00	0.03	0.978

	2	-0.06	-0.38	0.71	0.05	0.3.	0.76
	3	0.17	0.99	0.32	0.18	1.06	0.29
	1	-0.32	-1.07	0.29	0.06	0.21	0.83
Self-assessed shivering	2	-0.13	-0.41	0.68	0.03	0.11	0.91
	3	-0.41	-1.35	0.18	-0.18	-0.61	0.54
	1	0.21	1.30	0.20	0.17	1.06	0.29
$Comfort_T$	2	0.14	0.85	0.39	0.04	0.24	0.81
	3	0.32	1.94	0.05	0.29	1.79	0.07
	1	0.05	0.94	0.35	-0.02	-0.39	0.69
T_{skin}	2	0.05	1.07	0.29	-0.02	-0.43	0.66
	3	0.04	0.86	0.39	-0.04	-0.84	0.40
	1	-0.05	-0.24	0.81	0.06	0.30	0.77
DPG	2	0.09	0.46	0.64	0.12	0.59	0.56
	3	0.06	0.32	0.75	0.38	1.89	0.06
