# Supplementary material for: Abrupt light transitions in illuminance and correlated colour temperature result in different temporal dynamics and interindividual variability for sensation, comfort and alertness
Source: PLoS One. 2021 Mar 22;16(3):e0243259. doi: 10.1371/journal.pone.0243259 (PMC7984641; doi:10.1371/journal.pone.0243259)
Supplement: S1 File — The questions posed at the start of the session as control measures. (PDF) [file pone.0243259.s005.pdf]

Supporting information

S1. Start Questionnaire

How alert/sleepy do you feel at this moment?

1 (extremely alert) – 2 – 3 (alert) – 4 – 5 (neither alert, nor sleepy) – 6 – 7 (sleepy, but no difficulty remaining awake) – 8 – 9 (extremely sleepy, fighting sleep)

What transportation means did you use to come here?

Bike Car Train Walking

How long did it take you to get here?

…. Hours …. Minutes

How long (in total) have you been outside today (in daylight)

…. Hours …. Minutes

How many cups of coffee did you consume during the hour before the start of this session?

…. Cups

Did you consume any soda drink or energy drink during the hour before the start of this session?

Yes No

How many cups or glasses of caffeine containing drinks have you drunk today in total?

…. Cups/Glasses

How much did you eat during the hour before the start of this session?

Nothing A little bit Relatively a lot A lot
